# Platelets and Immune Responses During Thromboinflammation

**DOI:** 10.3389/fimmu.2019.01731

**Published:** 2019-07-26

**Authors:** Matthias Mezger, Henry Nording, Reinhard Sauter, Tobias Graf, Christian Heim, Nikolas von Bubnoff, Stephan M. Ensminger, Harald F. Langer

**Affiliations:** ^1^University Hospital, Medical Clinic II, University Heart Center Lübeck, Lübeck, Germany; ^2^DZHK (German Research Centre for Cardiovascular Research), Partner Site Hamburg/Lübeck/Kiel, Lübeck, Germany; ^3^Department of Cardiac Surgery, University Hospital Erlangen, Erlangen, Germany; ^4^Department of Hematology and Oncology, Medical Center, University of Schleswig-Holstein, Lübeck, Germany; ^5^Department of Cardiac and Thoracic Vascular Surgery, University Heart Center Lübeck, Lübeck, Germany

**Keywords:** platelets, innate immunity, complement, inflammation, stroke, infection, tissue remodeling, EAE

## Abstract

Besides mediating hemostatic functions, platelets are increasingly recognized as important players of inflammation. Data from experiments in mice and men revealed various intersection points between thrombosis, hemostasis, and inflammation, which are addressed and discussed in this review in detail. One such example is the intrinsic coagulation cascade that is initiated after platelet activation thereby further propagating and re-enforcing wound healing or thrombus formation but also contributing to the pathophysiology of severe diseases. FXII of the intrinsic pathway connects platelet activation with the coagulation cascade during immune reactions. It can activate the contact system thereby either creating an inflammatory state or accelerating inflammation. Recent insights into platelet biology could show that platelets are equipped with complement receptors. Platelets are important for tissue remodeling after injury has been inflicted to the endothelial barrier and to the subendothelial tissue. Thus, platelets are increasingly recognized as more than just cells relevant for bleeding arrest. Future insights into platelet biology are to be expected. This research will potentially offer novel opportunities for therapeutic intervention in diseases featuring platelet abundance.

## Introduction

In recent years, an increasing body of evidence demonstrates that platelets have several functions beyond hemostasis (1, 2). For instance, abundant evidence highlights the role of platelets for atherosclerosis, inflammation, and tissue regeneration. For example, platelets contribute to vascular inflammation of the brain during stroke or experimental autoimmune encephalitis (EAE) (2, 3). As a consequence, the question was raised whether platelets may even be considered as immune cells, despite the fact that they are not equipped with a nucleus (4). Most of the data available today originate from studies performed in small animal disease models and, thus, require validation in the course of the aforementioned diseases in patients. This is especially true in the context of immunology, since there are distinct differences between species (5). In this review, we seek to highlight the role of platelets for immune responses during thromboinflammation. In particular, we will address the relevance of platelet-associated mechanisms directly affecting the course of diseases in patients, as well as translational approaches.

## Platelets Originate From Megakaryopoiesis Located in the Bone Marrow

Like other hematopoietic cells, platelets are produced in the bone marrow (4). They are characterized by a small cell size and are missing a nucleus (4). Mean platelet volume in healthy individuals usually is in a range of 7 to 13 femtoliter (6). In the bone marrow, platelets derive from megakaryocytes. So far, one of the most important, but also best investigated factors known to be involved in megakaryopoiesis and platelet development is the cytokine thrombopoietin (TPO) (7). After binding of thrombopoietin to its counterreceptor Myeloproliferative Leukemia Virus Oncogene (c-MPL) on the megakaryocyte surface, intracellular signaling through Janus Kinase 2 (JAK2) is triggered (8, 9). The process of megakaryopoiesis is still incompletely understood, so far. For example, when thrombopoietin or the respective counterreceptor c-MPL is missing, the total amount of platelets is reduced to 10% of the normal platelet count (7). At the same time, platelet function as well as platelet morphology are not altered under these conditions (7) indicating that redundant factors besides thrombopoietin are involved in the process of terminal megakaryocyte maturation (10). Of note in myeloproliferative neoplasias (MPN), megakaryocytes are part of the malignant clone and source of inflammatory cytokines (11, 12), leading to chronic inflammation, constitutional symptoms and induction of fibroblast proliferation and bone marrow fibrosis (13) which are characteristic for primary and secondary myelofibrosis. In addition, this chronic inflammatory state might drive clonal evolution, cardiovascular disease and thrombohemorrhagic complications in these patients (14). The JAK1/2 inhibitor ruxolitinib attenuates inflammatory cytokines in myelofibrosis and clinical responses correlate with cytokine attenuation (15). Furthermore, there are hints pointing toward a role of megakaryocytes in antigen presentation through MHC-I leading to CD8 T-cell activation (16). Additionally, transfer of antigen loaded MHC-I from megakaryocytes to proplatelets has also been described (16). Vice versa, the inflammatory cytokine IL-6 was shown to be linked to increased plasma levels of thrombopoietin and an ultimately increased platelet number in a murine and human setting (17) and TPO was shown to augment platelet P-selectin (CD62P) expression stimulating platelet-leukocyte associations (18).

## Platelet Receptors and Interactions

**Figure 1:**
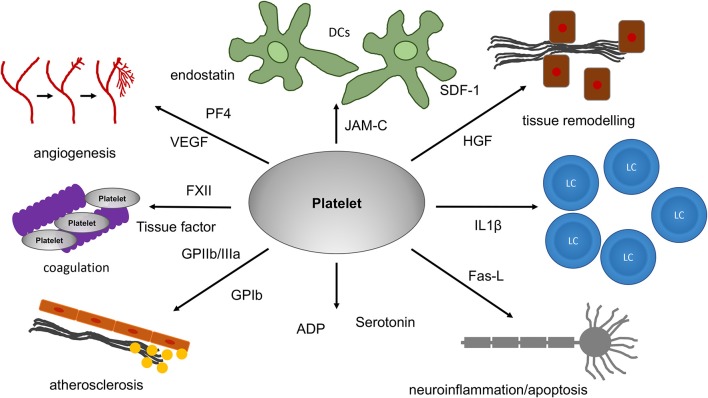
The role of platelets for tissue remodeling, apoptosis and angiogenesis. Platelets can have an influence on tissue remodeling under different pathological and physiological conditions. For tissue remodeling as well as apoptosis and angiogenesis, platelets are equipped with a multitude of receptors. HGF and SDF-1, for example, are of relevance in tissue fibrosis. In addition, platelets can release a multitude of proteins. Among these are proteins with strong pro- or antiangiogenic effects. PF4 and endostatin are known to inhibit angiogenesis. In contrast, VEGF, which platelets can release as well, strongly increases angiogenesis. Since platelets are transported within the blood-stream they can reach almost all organs and tissues thereby even influencing processes associated with inflammation in the brain and systemic diseases like atherosclerosis. In addition, platelet mediated apoptosis through FasL on the platelet surface has been reported in the brain. VEGF, vascular endothelial growth factor; PF4, platelet factor 4; SDF-1, stromal derived factor; HGF, hepatocyte growth factor; JAM-C, junctional adhesion molecule C; IL1β, Interleukin 1β; FasL, Fas-ligand; ADP, adenosine diphosphate; FXII, coagulation factor XII.

**Figure 2:**
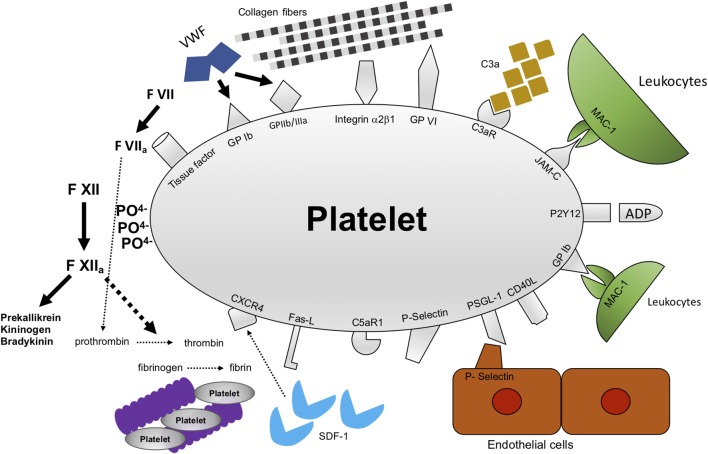
Mechanisms of interactions between platelets and their microenvironment. Platelets were first recognized to be important for thrombosis and hemostasis after vessel injury has happened. In addition, there is accumulating evidence that platelet function goes beyond thrombosis and hemostasis. Platelets interact with a multitude of cells and proteins. Among these are receptors and proteins modulating thrombosis/hemostasis, e.g., GPIbα, GPIIb/IIIa, GPVI, and polyphosphates. In addition, platelets modulate inflammation, e.g., through C3aR, JAM-C, PSGL-1, P-selectin, CXCR4. GPIbα, glycoprotein Ibα; GPIIb/IIIa, glycoprotein IIb/IIIa; GPVI, glycoprotein VI; C3AR, receptor for complement factor 3; JAM-C, junctional adhesion molecule C; CXCR4, C-X-C-chemokine receptor type 4.

**Figure 3:**
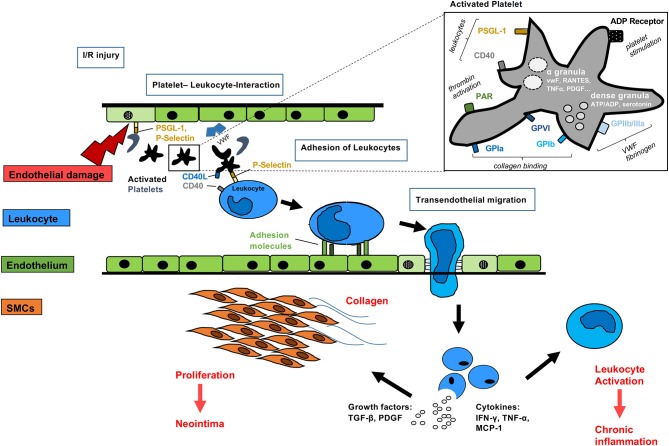
Mechanisms of activated platelets after organ transplantation Platelets may be activated before, during and after allogeneic organ transplantation. Direct interaction with the immune system may occur via simultaneous P-selectin and CD40 binding to leukocytes. Non-nucleated platelets contain dense granules with serotonin, ADP and α-granules with pro-inflammatory cytokines, such as RANTES, TNFα, PDGF, and VWF. Surface receptors including glycoprotein receptors lead to collagen binding, VWF and fibrogen activation. Activated leukocytes adhere to the endothelium of transplanted grafts, migrate and release growth factors and pro-inflammatory cytokines finally leading to smooth muscle cell proliferation, collagen deposition and further leukocyte activation.

Despite being small particles, platelets are equipped with a multitude of receptors to interact with themselves, with other cells, e.g., endothelial cells and cells of the immune system and, of course, with the extracellular matrix (Figure 1). In general, four types of receptors can be found on platelets: integrins, glycoproteins, selectins and receptors of the immunoglobulin type (Figure 2). First, after injury to the vessel wall has occurred, GPIbα on the platelet surface binds von Willebrand factor (VWF). This is especially important under conditions of high shear stress such as in the arterial branch of the vascular system (19). In addition, platelets bind to exposed subendothelial collagen fibers through glycoprotein VI (GPVI) (20–22), finally leading to a high affinity state of GPIIb/IIIa (23) (Figure 2). Subsequently, fibrinogen can be bound to GPIIb/IIIa on the platelet surface thereby crosslinking platelets with platelets and platelets with endothelial cells (24). Inhibiting GPIIb/IIIa was shown to be beneficial in myocardial infarction (25), however in stroke, clinical studies revealed an increased risk of bleeding (26). Ongoing thrombus formation is further supported by interaction of integrins on the platelet surface with fibrinogen and components of the extracellular matrix such as collagen and laminin. Interaction with fibrinogen is mediated through integrin α5β1 on the platelet surface, binding to laminin is mediated through integrin α6β1 (27). In addition, binding to collagen also involves integrin α2β1, which brings about platelet filopodia and lamellipodia formation (28). After platelet activation has happened, mediators from platelet granules are released further fueling platelet activation (29). Among these are P-selectin, VWF and fibrinogen from α-granules and ADP, calcium and serotonin from dense granules (30–32). Selectins were shown to be involved in platelet-endothelial and platelet-leukocyte interactions as well especially under conditions when the endothelial barrier is not disrupted (Figure 3). For instance, in ischemia-reperfusion injury platelets were shown to be involved in leukocyte recruitment since both adherent leukocytes as well as emigrated leukocytes were significantly reduced when either platelets were depleted through administration of platelet depleting serum or platelet receptors were blocked through administration of blocking antibodies (33). Besides platelets, P-selectin as well as VWF are stored within Weibel-Palade bodies in endothelial cells, too (34, 35). P-selectin on platelets was shown to interact with PSGL-1 expressed on leukocytes (36). The interaction of P-selectin on platelets with PSGL-1 was also demonstrated for platelet-neutrophil interactions (37, 38). In addition, platelets can interact with endothelial cells through binding of GPIbα on platelets to P-selectin expressed on endothelial cells (39). Under conditions of inflammation such as in atherosclerotic lesions, platelet-mediated recruitment of leukocytes through platelet P-selectin has been shown to be followed by platelet binding with CXCR3 to inflamed endothelium expressing CX3CL1 (40).

Another mechanism especially relevant for the recruitment of dendritic cells to the vascular wall was shown to be mediated by JAM-C (Figure 2), a member of the immunoglobulin family of receptors expressed on platelets, and MAC-1 on dendritic cells (41). Interaction of MAC-1 with platelets bound to endothelial cells has been shown for endothelial transmigration of neutrophils, too (42). Both the interaction of platelets with neutrophils as well as the interaction of platelets with DCs initially requires binding of platelet P-selectin to PSGL-1 on leukocytes (41, 42). Interestingly, MAC-1 was shown to be a binding partner of platelet GPIbα, too (43). Fascinatingly, MAC-1 deficiency goes along with delayed thrombosis although hemostasis seems not to be affected (43). This raises the question whether targeting MAC-1 could offer a way to efficiently inhibit thrombosis without hampering with hemostasis.

Finally, platelets were shown to be involved in the recruitment of CD34^+^ bone marrow cells and bone marrow progenitor cells to sites of vascular injury (44), linking platelets to tissue remodeling and neointima formation. In this context, platelet P-selectin as well as GPIIb/IIIa were shown to be involved (44). Blockade of platelet receptors with monoclonal antibodies abrogated recruitment of CD34^+^ cells to sites of vascular injury, further underlining the relevance of platelets for recruitment of bone marrow cells to the vascular wall (44).

## Platelets are Closely Linked to the Plasmatic Coagulation System, Thereby Linking Hemostasis and Thrombosis to Inflammation

Platelets mediate thrombosis and hemostasis through the different receptors expressed on the platelet surface but also through soluble mediators released immediately after platelet activation. With respect to hemostasis, platelets operate in parallel to the plasmatic coagulation cascade. In a recent review, the ongoing debate on whether platelets can release tissue factor or not is delineated in a concise fashion (45). Some studies show that platelets can release tissue factor by themselves after activation (46), for instance on the surface of platelet microparticles (47). Tissue factor initiates the extrinsic coagulation cascade (48, 49). On top of that, procoagulant activity was significantly reduced after either tissue factor or F VII were missing in thrombin-activated platelets adhering to fibrinogen (46). Accordingly, in the presence of anti-tissue factor antibody an increased time was observed for clot formation (46). In contrast, other studies were not able to show a role of platelets for tissue-factor mediated coagulation. One study could only show enhanced tissue factor expression when monocytes were present besides platelets, too (48). In another study, no relevant tissue factor secretion could be detected after prolonged stimulation of platelets with lipopolysaccharides (LPS) (50). In the same study, stimulation of a monocyte cell line with LPS yielded large amounts of tissue factor leading to clot formation through activation of the extrinsic coagulation cascade (50), indicating that monocytes in contrast to platelets are responsible for tissue factor production.

There is also evidence that platelets interact with the intrinsic pathway of the coagulation system (Figure 2). Platelets contain polyphosphates that can be externalized onto the platelet membrane, thereby creating a large surface with negative charge triggering activation of coagulation factor XII (contact system of the coagulation cascade) (51). After activation, FXII activates FXI thereby further accelerating coagulation. Additional investigations regarding the role of FXII for coagulation revealed that FXII is necessary for stable thrombi in different models of arterial injury in mice (52). Coagulation Factor XII, besides mediating coagulation, seems to have a role for driving inflammation, as well. After activation, FXII drives activation of the prekallikrein-kininogen-bradykinin-cascade whereby inflammation is triggered (53). Strikingly, in humans FXII deficiency is not associated with an increased bleeding risk. In contrast, in a study population of 74 patients suffering from FXII deficiency, two subjects had already suffered venous thromboembolism at an age <40 years (54). In an experimental design where FXII knockout mice as well as wildtype mice were subjected to transient middle cerebral artery occlusion (tMCAO), FXII knockout mice showed similar reduction in cerebral blood flow in MRI measurements 2 h after tMCAO (55). However, 24 h after tMCAO cerebral blood flow was markedly improved in FXII knockout mice compared to their wildtype counterparts (55). In addition, in tMCAO intravascular fibrin deposits leading to vessel occlusion were reduced in FXII knockout mice. In wildtype animals however, occluding thrombi contain both platelets and fibrin suggesting synergism between FXII mediated fibrin formation and platelets (56). Indeed, knockout mice for FXII showed impaired platelet rich occlusive thrombi in distinct arterial beds (57). This finding was also reported to be of particular relevance in the context of neurovascular inflammation since mice lacking FXII showed protection from ischemic brain damage (55).

## Platelets, Despite Lacking a Nucleus, Exhibit Translational Activity and Can Release a Multitude of Active Factors

Early experiments in the 1960 by Warshaw et al. produced first evidence on protein synthesis by platelets since they could inhibit translational activity in platelets with puromycin (58). However, it took several decades and the power of PCR technology and proteomics to demonstrate that platelets are capable of protein synthesis since they incorporate RNA as well as a transcriptional and translational machinery (59). Interestingly, platelets can splice intronic interleukin-1β pre-mRNA generating fully mature interleukin-1β mRNA and interleukin-1β protein (60). Interleukin 1 comprises a group of 11 cytokines with both pro- and anti-inflammatory effects and is a good example demonstrating the role of platelets at an intersection between inflammation and thrombosis (61) (Table 1). The release of a multitude of factors from platelets makes them powerful tools for interaction with various cells and tissues (Figure 1, Table 1). Platelet proteomics could reveal that more than 300 proteins are secreted from human platelets after they become activated by thrombin (62). Among these were proteins responsible for coagulation including the factors FV or FXIII (62), but also proteins such as albumin, platelet factor 4 (PF4, CXCL4) or matrix metalloproteinase inhibitor 1 (62). Finally, factors responsible for tissue remodeling such as CXCL12 (SDF-1) and growth factors, for example hepatocyte growth factor (HGF), can be released by platelets (63, 66). In addition, a specialized way of how translational activity in platelets is modulated through altered ribosome function has been uncovered. It was observed that the platelet lifespan was linked to mRNA and ribosomal RNA content, both of which were reduced with increasing platelet age (78). A recent study could show that loss of the RNA surveillance factor Pelota is a decisive mechanism by which platelets stop decay of their limited pool of mRNA (79). This finding seems to be of relevance in a clinical context, too, since an association of platelet protein synthesis and cardiovascular disease could be demonstrated. Platelet WDR-1 (WD-40 repeat domain 1) mRNA and protein level was found to be significantly lower in patients suffering from cardiovascular disease compared to matched controls (80). WDR-1 is known to enhance actin depolymerizing factor activity (81), thereby altering the platelet cytoskeleton (82). In addition, WDR-1 mutations in mice were linked to defects in neutrophil cytoskeleton and defective platelet production and, finally, brought about macrothrombocytopenia and autoinflammation (83). Whether the autoinflammation observed in this model is attributable only to defective neutrophil function or if platelets are involved as well, e.g., through altered platelet-neutrophil interactions, still remains to be investigated in the future.

**Table 1:** Receptors and factors associated with platelets relevant for thromboinflammation.

**Receptor/Factor**	**Relevant for**	**References**
IL1β	Inflammation	(60, 61)
PF4	Tissue remodeling/angiogenesis	(62)
CXCL12(SDF)	Tissue remodeling	(63–65)
HGF	Tissue remodeling	(66, 67)
tissue factor	Coagulation	(46, 47)
polyphosphates/FXII	Coagulation/thromboinflammation	(51)
GPIbα/VWF	Coagulation/atherosclerosis/neuroinflammation/thromboinflammation	(19, 68, 69)
GPIIb/IIIA/fibrinogen	Coagulation/atherosclerosis/neuroinflammation	(19, 68, 69)
GPVI/collagen	Coagulation	(20, 21)
ADP	Coagulation	(70)
thromboxane A2	Coagulation	(70)
serotonin	Coagulation/inflammation	(71, 72)
P-selectin	Inflammation/atherosclerosis/thromboinflammation	(36, 44)
PSGL-1	Inflammation	(36)
JAM-C	Inflammation/apoptosis	(41)
C3aR	Inflammation/atherosclerosis/thrombosis	(73, 74)
C5aR	Atherosclerosis	(75)
VEGF	Tissue remodeling/angiogenesis	(76)
endostatin	Tissue remodeling/angiogenesis	(76)
FAS-L	Tissue remodeling/apoptosis	(77)

## Platelets Interact With Cells of the Innate Immune System and Endothelial Cells Making Them Important Players Involved in Sepsis and Inflammation-Induced Bleeding

When inflammation takes place, endothelial cells lining the inner surface of blood vessels become activated and change their receptor expression profile and their phenotype. Platelets as well as VWF were shown to be closely linked to inflammation in a neurovascular setting (84). For instance, antibody-mediated blockade of platelet GPIbα, the receptor for VWF, after induction of tMCAO was associated with improved cerebral blood flow in magnetic resonance imaging (85) leading to improved outcome (86). In addition, deficiency in VWF was paralleled with a protective phenotype in tMCAO, as well (87). In contrast, when VWF was reconstituted in VWR knockout mice, protection from cerebral ischemia was diminished (87). A common finding in severe generalized infections and sepsis is that platelet count is markedly reduced (88). In addition, a prolonged time span of thrombocytopenia is correlated with increased mortality in intensive care patients (88). Clinical data, although derived from retrospective studies, indicate that treatment with antiplatelet drugs is beneficial under conditions of severe inflammation/sepsis raising the question of how platelets might be involved in sepsis (89, 90).

Interestingly, in thrombocytopenia inflammatory mediators like LPS increased the risk of bleeding (91), underlining the importance of platelets not only for thrombosis and hemostasis but also for inflammation. This finding was demonstrated in various models of inflammation in mice. For instance, when mice were suffering from thrombocytopenia, induction of contact dermatitis resulted in increased bleeding compared to WT animals (91). Investigations using the dorsal skinfold chamber model, which enables *in vivo* observations of the vascular system, further confirmed the relevance of inflammation for bleeding in cases of thrombocytopenia (91). Mice suffering from severe thrombocytopenia as well as inflammation induced by application of LPS showed spontaneous intraalveolar hemorrhage (91). In addition, inflammatory bleedings under conditions of thrombocytopenia in the skin as well as in the lungs, were shown to be strongly dependent on neutrophil-endothelial interactions, thereby involving endothelial VE-Cadherin (92). In addition, platelet GPVI was shown to attenuate inflammation-induced bleeding mediated through neutrophils by binding to exposed subendothelial collagen (93). Recently, the maintenance of inflammatory hemostasis was shown to be organ- and stimulus-dependent, i.e., GPVI and GPIIb/IIIa were not required for prevention of intraalveolar bleeding after LPS challenge, while GPIbα attenuated inflammation-induced bleeding in the lung (94).

However, in sepsis adverse outcome is not only due to inflammation-induced bleeding. Instead, increased mortality could be observed irrespective of bleeding in cases when thrombocytopenia was present (95). Here, platelet transfusion was shown to be beneficial with respect to sepsis (95). The mechanisms behind the protective role of platelets observed, involved prostaglandin E2 biosynthesis within platelets through COX-1, and activation of prostaglandin receptors on macrophages (95). Furthermore, platelets have been demonstrated to protect from extracorporeal-circulation induced inflammatory lung injury. Here, platelet transfusion was associated with milder lung injury which also went along with decreased levels of TNFα and neutrophil elastase which were measured in the blood plasma (96). Blocking platelet GPIIb/IIIa through administration of Tirofiban reversed the observed effects (96). Finally, platelets were shown to limit neutrophil-induced endothelial damage by interaction with neutrophil elastase, as well (93).

Furthermore, the interplay of DCs and platelets was shown to be of paramount importance with respect to bacterial infections. Listeria monocytogenes is a bacterial infection threatening immunocompromised individuals as well as creating fetal infections that finally can lead to abortion (97, 98). Recently, new insights on the role of platelets for infection with Listeria monocytogenes were published. Platelets, with the aid of complement C3 and GPIbα, were shown to be directly involved in the transport of Listeria to splenic CD8α+ DCs, which then cross-present antigenic peptides via MHC-I to T-helper cells thereby creating an adaptive immune response (99). Besides Listeria, C3-mediated platelet association could be demonstrated for other gram-positive bacteria, e.g., Staphylococcus aureus (99). This further underlines the importance of platelets for any defense against bacteria and the development of adaptive immunity. In addition, blockade of GPIIb/IIIa had protective effects when sepsis was induced in mice (100). Furthermore, when GPIIb/IIIa was blocked, reduced levels of apoptosis in splenocytes in an *in vitro* approach were observed (100).

Another link of platelets to inflammation could be demonstrated *in vivo* in a mouse model of immune complex-mediated systemic shock, since interaction of platelets with immune complexes led to serotonin release from platelets (71) (Table 1). After platelet depletion, injection of immune complexes did not create a systemic shock-reaction according to the clinical scores (71). Further investigations of the mechanisms underlying platelet response to immune complex-mediated shock revealed that GPIIb/IIIa is necessary in this context since dysfunctional receptor ligand interaction due to mutated fibrinogen abrogated immune complex-mediated systemic shock (71). In contrast, neither P-selectin nor GPIbα were required for immune complex-mediated shock (71).

## Platelets Interact With the Complement System

The complement system is a very old and well-conserved cascade of proteases produced in the liver, which are involved in clearance of dead cells as well as pathogens that have passed the natural barrier between the body and the environment (101, 102). The complement system is involved in various human pathologies associated with dysregulated platelet function and disseminated thrombosis in microvessels, e.g., hemolytic uremic syndrome (HUS) (103). In atypical HUS (aHUS), deposition of complement factors C3 and C9 could be verified on the platelet membrane (104). Furthermore, CD40L expression was increased on platelets, indicating platelet activation (104). Complement receptors for C3a and C5a are expressed on platelets, too (73, 75) (Table 1). Interestingly, in HUS renal dysfunction is caused through microthrombosis in the renal vascular system and mutations in complement C3 were shown to predispose to development of aHUS (105). Investigating the role of platelets for cardiovascular disease, we were able to show in a clinical study with patients suffering from coronary artery disease that expression of complement receptor for C5a on platelets (C5aR) could be correlated to markers of platelet activation (75). Interestingly, *in vitro* investigations of platelets with flow cytometry performed after platelets had been stimulated with oxidized low-density lipoprotein (oxLDL) revealed that expression of C5aR and P-selectin increased after platelet incubation with oxLDL (75). In the same study, an inverse correlation between platelet bound oxLDL and plasma C5a could also be observed (75). Another study performed in this field uncovered a strong correlation between expression of C3aR and GPIIb/IIIa on human platelets with known coronary artery disease (73), further highlighting the intimate connection between the complement system and platelets. When thrombi of cardiovascular patients were analyzed, coexpression of C3aR and GPIIb/IIIa was evident (73). Additional investigations *in vivo* in a mouse model deficient for either C3aR or C3 revealed that C3a affects not only bleeding time but also tissue injury after stroke, myocardial infarction and thrombosis (73). Reconstitution of mice deficient for C3aR with WT platelets, could reverse the observed protective effects of C3aR deficiency with respect to thrombosis-related ischemic injury (73). Bleeding was aggravated in the C3aR knockout mice, which could be reversed after transfusion of WT platelets (74). In addition there is increasing evidence that platelet P-selectin could be a receptor for C3b, underlining the close intersection between coagulation and inflammation (106). The crosstalk between platelet activation and the complement cascade is a good example of how closely platelets link inflammation to thrombosis and vice versa.

## The Role of Platelets for Tissue Remodeling, Apoptosis and Angiogenesis

### The Role of Platelets for Tissue Remodeling

Besides the established function of platelets for coagulation, there is also increasing evidence for a function of platelets in tissue remodeling and angiogenesis (Figure 1, Table 1). For example, platelets are directly involved in the process of atherosclerosis, even before any thrombotic event. Massberg et al. could demonstrate in a mouse model of ApoE deficient mice suffering from severe atherosclerosis, that the development of atherosclerotic lesions was preceded by platelet adhesion to the endothelium through interaction of GP Ibα and GPIIb/IIIa with the arterial wall (68). Platelet adhesion was the first event preceding atherosclerotic plaque formation, followed by leukocyte adhesion to the vascular wall (68). There is conclusive evidence supporting a role of platelets not only for acute atherothrombosis, for instance in myocardial infarction, but also in the process of chronic vascular inflammation. For example, individuals suffering from familial hypercholesterolemia display elevated levels of platelet microparticles in the blood (107). These microparticles exhibit markers of platelet activation such as P-selectin or GPIIb/IIIa and tissue factor (107). Furthermore, MRI-imaging performed in these patients revealed increased atherosclerotic plaque burden reflecting dangerous lipid-rich cores prone to rupture, particularly in the case when tissue factor bearing microparticles were present (107). Increased levels of platelet microparticles were also reported in patients suffering from severe heart failure requiring cardiac assist device therapy (108). These patients often suffer both from bleeding disorders as well as thromboembolic complications (109) and management of coagulation is a major concern.

As already mentioned, platelets can release various factors already known from tissue remodeling processes (Figure 1). Among those factors released from platelets are CXCL12 and hepatocyte growth factor (HGF), respectively (44, 63, 66) (Table 1). HGF is highly relevant in tissue fibrosis and remodeling as investigated in an *in vivo* model, in which Syrian hamsters suffering from hereditary cardiomyopathy were treated with HGF (67). The animals showed severe cardiac dysfunction and fibrosis. After treatment with recombinant HGF for 3 weeks, cardiac fibrosis was ameliorated (67). This was accompanied by reduction of transforming growth factor β 1 (TGFβ1) and type I collagen (67). Interestingly, platelets themselves were shown to interfere with HGF, as well, since they could inhibit migration of mesenchymal stem cells to apoptotic cardiomyocytes. HMGB-1 released by platelets was directly involved in this process (110, 111). However, the inhibitory role of platelets for recruitment of stem cells has also been questioned since an inhibitory effect on recruitment of vascular endothelium has been reported, too (112).

CXCL 12 also known as SDF-1, which can be released by platelets, is involved in neointima formation after vascular injury through recruitment of vascular smooth muscle cell progenitors (64, 65). First, SDF-1 is released by media smooth muscle cells undergoing apoptosis after injury has happened (65). Subsequently, SDF-1 binds to platelets which then are attached to the vessel wall at sites of injury (65). This is followed by attachment of smooth muscle cell progenitor cells to the platelets mediated by P-selectin and CXCR4, the SDF-1 counterreceptor (65). With respect to cardiovascular disease it has been demonstrated that platelet expression levels of SDF-1 correlate with adverse outcomes (113).

Platelets were shown to be involved in experimental autoimmune encephalitis (EAE) as well, linking them to another field of neuroinflammation and tissue remodeling besides stroke (69, 114). Experimental autoimmune encephalitis is a preclinical model for the human disease multiple sclerosis (MS), during the course of which inflammation of the brain is induced through administration of central nervous tissue or myelin peptides (115). Both in the human as well as in the murine disease, platelet specific CD41 was shown to be upregulated in brain tissue (69, 116). In addition, the course of the disease was affected as a result of platelet depletion. When platelets were depleted through administration of platelet depleting serum in the effector phase of the disease, reduced microgliosis within inflamed brain tissue could be observed. Both interfering with platelet GPIbα as well as platelet GPIIb and the GPIbα counterreceptor on leukocytes, MAC-1, through administration of blocking antibodies was able to ameliorate EAE (69). Recently, the importance of the timing of platelet depletion for the course of the disease was further supported by another study (114). Platelet depletion in the immunization phase of EAE did not have an impact on the course of the disease (69, 114). Microarray analysis of the spinal cords after induction of EAE revealed several factors relevant for inflammation such as CCL2, CCL5, CXCR4, and IL1β which were downregulated significantly after platelet depletion (69). The question whether platelets themselves, are drivers of neuroinflammation in the context of experimental autoimmune encephalitis, or whether they contribute to experimental autoimmune encephalitis through recruitment of inflammatory leukocytes either by receptor-ligand interaction with leukocytes or by releasing inflammatory mediators is still open. Hopefully, future research will be able to resolve this question thereby significantly improving therapies for patients suffering from multiple sclerosis.

### The Role of Platelets for Angiogenesis

Angiogenesis in general is a tightly regulated process that is modulated by a multitude of cells and soluble factors (117, 118). Angiogenesis can be beneficial, for instance with respect to wound healing and tissue regeneration (117, 118). However, angiogenesis can also be harmful since tumors need a so-called angiogenic switch to grow beyond a certain size (117, 118). Angiogenesis is tightly associated with inflammation (119). Some of the proteins released from platelets possess angiogenic potential either exerting pro- or antiangiogenic responses on endothelial cells (Table 1). Experimental approaches using Matrigel, an extracellular matrix from the Engelbrecht-Holm-Swarm (EHS) sarcoma, together with endothelial cells is an established way to investigate angiogenesis *in vitro* (120). In a Matrigel model, a proangiogenic effect of platelets could be demonstrated after endothelial cells and platelets had been added (121). Interestingly, platelets directly adhere to endothelial cells (121). Adding platelet supernatant to the Matrigel showed significantly reduced tube formation compared to adding platelets (121), further supporting that direct platelet-endothelial interaction is necessary for the observed proangiogenic effect *in vitro*. Regarding platelet physiology, differential release of pro- or antiangiogenic factors happens depending on the stimulus (122). There are hints that ADP as well as GPVI favor a proangiogenic phenotype of platelets (122). In addition, platelets can release VEGF after stimulation with ADP (76). In contrast, PAR-4 favors an antiangiogenic phenotype of platelets (122). This was also observed, when platelets were stimulated with thromboxane A2 since this triggered release of the antiangiogenic agent endostatin (76). Platelets have been recognized as a major source of vascular endothelial growth factor (VEGF) (123), one of the most important growth factors involved in angiogenesis (124). When VEGF is released, it can bind to a variety of growth factor receptors thereby directing proangiogenic effects (124). After VEGF has bound to endothelial cells, they start to proliferate and to form tubes, which results in the formation of new vessels with recruitment of pericytes as well as smooth muscle cells (124).

Furthermore, platelets have also been implicated in ischemia-induced revascularization after arterial occlusion, which is primarily achieved by arteriogenesis (125). Platelets have been shown to recruit bone marrow-derived cells in response to ischemia in mouse models of hindlimb ischemia or tumor implantation in mice (126). After platelet depletion through administration of an anti GPIbα antibody, levels of bone marrow cells within the tissue were significantly reduced (126). Among the different factors mediating platelet function, α-granules as well as the antiangiogenic protein thrombospondin were shown to be relevant for the observed effects (126). In addition, a role of platelet microparticles for angiogenesis has been reported, too (127).

These effects may be the reason why platelet-rich plasma (PRP) is a potent agent to foster wound healing. Using PRP in a patient collective suffering from dehiscent sternal wounds or necrotic skin ulcers has been shown to be beneficial since duration of hospital stay after administration of PRP was almost half of the time in patients with dehiscent sternal wounds compared to standard care (128). Unfortunately, detailed investigations of the underlying mechanisms were not undertaken, so far, and we are left with speculating on the molecular mechanisms involved. Nevertheless, a COCHRANE review has confirmed the clinical benefit of the administration of platelet-rich plasma in patients with diabetes and chronic wounds (129), offering promise for future platelet-based therapies in this field.

### The Role of Platelets for Apoptosis

Recently, there were hints from preclinical studies in a murine model that platelets may be involved in the process of apoptosis (Figure 1, Table 1). Apoptosis in general can be induced both through external signaling as well as through internal pathways (130). The external pathway involves several factors (TNFα, FasL, TRAIL), which after binding to their respective receptor activate an intracellular signaling cascade finally leading to activation of a set of specialized enzymes the so-called caspases (130). Besides the external pathway there is an intrinsic way how apoptosis can be initiated as well. The intrinsic pathway relies on cytochrome c which activates caspases after their release from mitochondria (130). Finally the cells undergo a special program which finally leads to cellular clearance (130). Schleicher et al. could demonstrate that platelets can be found in the brain tissue after experimental stroke in the tMCAO stroke model. Apoptosis in the brain tissue was reduced after platelet depletion (77). Similar observations were made when GPIbα deficient mice were used (77). Further analysis revealed that platelets express FasL in their membrane thereby mediating apoptosis (77). In contrast, Bax/Bak signaling of the internal pathway of apoptosis was not required but additionally contributed to apoptosis (77). A previously unrecognized role of platelets for apoptosis was also identified in the context of platelet-DC interactions. JAM-C was shown to be directly involved in platelet-DC interactions mediating apoptosis of DCs (41). Platelets were directly responsible for the recruitment of DCs to the vessel wall. *In vivo*, when no vascular lesion was present in a model of carotid artery injury, no DCs were recruited to the vessel wall. In contrast, after vascular injury, the number of DCs adhering to the vessel increased markedly (41).

Finally, patients suffering from human immunodeficiency virus (HIV) were shown to have an increased rate of cardiovascular events (131), despite having achieved stable disease by means of combined antiretroviral therapy. In addition, *in vitro* investigations could show that markers of platelet activation, e.g., P-selectin were upregulated under this condition (132). Furthermore, activation of the intrinsic pathway of apoptosis was more prevalent in platelets from patients suffering from HIV infection despite being under viral control (132), suggesting dysregulated platelet function as one possible contributing factor to increased numbers of cardiovascular events. This was further underlined in a HIV positive patient collective suffering from acute coronary syndrome where, despite receiving Aspirin and P2Y12 inhibitor therapy, high residual platelet reactivity could be measured (133). However, the mechanism underlying the observed dysregulated platelet function in patients suffering from HIV is incompletely understood, yet. Altogether, the data reported strongly point to an intimate connection of platelets and inflammation and a function of platelets beyond thrombosis and hemostasis.

### Platelets and Cancer

A common finding is that cancer is associated with thrombosis and embolism (Trousseau phenomenon). The increased risk of thrombosis in cancer can be attributed to a variety of mechanisms, including increased expression and release of procoagulant factors and microparticles by tumor cells and platelets, abnormal tumor vascularity and increased inflammation [reviewed in (134)]. An increasing body of evidence indicates that, in addition to increasing the risk of thrombosis, platelets can also contribute to tumor progression and metastasis by altering the tumor microenvironment, by expression of growth factors and proangiogenic factors and by assisting neoplastic cells to evade apoptosis (76, 135–137). In addition, platelets can promote metastasis by protecting tumor cells in circulation from immune surveillance and by assisting tumor cell adhesion and transmigation of the vascular endothelium (138, 139). Investigations in the field of platelets and cancer have shown that induction of thrombocytopenia by platelet-depleting antibodies increased the efficacy of paclitaxel therapy in a murine model of breast cancer, likely through increased tumor vascular permeability (140). In addition, platelet depletion caused intratumor hemorrhage in different tumor models in mice (141). Interestingly, no increase in intratumor hemorrhage could be observed when GPIbα was blocked (141). However, as expected, tail bleeding time was markedly increased after blockade of GPIbα (141). In patients suffering from ovarian cancer, the patients' platelet count had a prognostic relevance. Thrombocytosis was associated with reduced overall survival and resistance to chemotherapy (142, 143). Although these results point to a central role of platelets in cancer biology, these insights did not translate to therapeutic strategies exploiting the function of platelets in cancer progression and metastasis, so far. While the use of low-molecular-weight heparin has been demonstrated to reduce the rate of recurrent thrombosis and thus represents the current standard in patients with cancer who suffered venous thromboembolism (144), the use of antiplatelet therapies in patients with cancer remains controversial. Although retrospective analyses suggest protective effects of daily aspirin for some cancers (145), antiplatelet drugs have not entered cancer therapy.

### Platelets Participate in Vascular Remodeling After Organ Transplantation

During the process of solid organ transplantation, platelets can be activated at multiple points. Activation and subsequent degranulation may already occur during graft procurement in organ donors (146). Brain death boosts a catecholamine storm resulting in organ malperfusion (147). Furthermore, blood or platelet transfusions administered perioperatively can promote platelet activation. Prolonged ischemia during organ procurement or transplantation results in platelet activation via P-selectin and CD40L (148, 149). Platelet activation in organ recipients can occur in patients with preexisting diseases such as atherosclerosis (150) or in contact with bioincompatible surfaces such as in dialysis patients, patients with a ventricular assist device and, of course, through contact with surfaces during extracorporeal circulation at time of transplantation (151, 152). Subsequently, activated platelets may trigger an inflammatory reaction of endothelial cells and interfere with leukocytes resulting in cellular rejection (153, 154) (Figure 3). These processes may lead to the development of cardiac allograft vasculopathy and are the basis for a series of experiments regarding the role of platelets for chronic rejection after heart transplantation (155, 156). The experimental therapeutic approach of platelet inhibition with the P2Y12 ADP receptor blocker clopidogrel especially in combination with an mTOR inhibitor was very effective in a mouse aortic transplantation model, where allograft vasculopathy was almost abolished (157, 158). These findings paved the ground for a multi-center clinical trial called CEDRIC. However, the CEDRIC trial (Clopidogrel add on Certican: Effects on Coronary Diameter Reduction and Intimal Hyperplasia in Long-term follow-up after Cardiac Transplantation) had to be terminated due to recruiting problems and therefore further studies are necessary to ultimately validate this concept for a broad clinical application. Taken together, platelets play an important role in vascular remodeling after organ transplantation through both antithrombotic properties and the above-mentioned immune modulatory effects (Figure 3).

## Concluding Remarks

Taken together there is increasing evidence for a role of platelets beyond hemostasis and thrombosis. Platelets are closely connected to inflammation. Contextual examples for this intimate connection between platelets and thromboinflammation are the plasmatic coagulation system as well as the complement system. Nonetheless, a lot of questions are still unanswered. One such question is how the beneficial effect of FXII deficiency on ischemic stroke can be explained. Solving this question might point the way how the outcome of this—sadly—very often disabling disease might be improved for patients. Another promising field of research is the close connection of the complement system and platelets to diseases featuring disseminated thrombosis, e.g., hemolytic uremic syndrome. Effective therapies for HUS are still missing, which raises the question, whether a clinically beneficial resolution of thrombus formation can be achieved by modulation of platelet function. Future research in platelet biology has the potential to show us even more novel, previously unexpected ways how platelets are directly involved in the most fundamental processes of health and disease.

## Author Contributions

MM, HN, RS, TG, CH, NvB, SE, and HL wrote parts of the manuscript. HL conceptualized and submitted the manuscript.

### Conflict of Interest Statement

The authors declare that the research was conducted in the absence of any commercial or financial relationships that could be construed as a potential conflict of interest.
